# Validity of urges to smoke measures in predicting smoking relapse during treatment in primary care

**DOI:** 10.1038/s41533-021-00259-3

**Published:** 2021-12-09

**Authors:** Daniel Kotz, Carolien van Rossem, Wolfgang Viechtbauer, Mark Spigt, Onno C. P. van Schayck

**Affiliations:** 1 Institute of General Practice, Addiction Research and Clinical Epidemiology Unit, Medical Faculty of the Heinrich-Heine-University Düsseldorf, Düsseldorf, Germany; 2 Department of Family Medicine, CAPHRI School for Public Health and Primary Care, Maastricht University, Maastricht, The Netherlands; 3 Research Department of Behavioural Science and Health, Institute of Epidemiology and Health Care, University College London, London, United Kingdom; 4 Department of Psychiatry and Neuropsychology, School for Mental Health and Neuroscience, Maastricht University, Maastricht, The Netherlands; 5 General Practice Research Unit, Department of Community Medicine, The Arctic University of Tromsø, Tromsø, Norway

**Keywords:** Epidemiology, Preventive medicine

## Abstract

In the context of smoking cessation treatment in primary care, identifying patients at the highest risk of relapse is relevant. We explored data from a primary care trial to assess the validity of two simple urges to smoke questions in predicting long-term relapse and their diagnostic value. Of 295 patients who received behavioural support and varenicline, 180 were abstinent at week 9. In this subgroup, we measured time spent with urges to smoke (TSU) and strength of urges to smoke (SUT; both scales 1 to 6 = highest). We used separate regression models with TSU or SUT as predictor and relapse from week 9–26 or week 9–52 as an outcome. We also calculated the sensitivity (SP), specificity and positive predictive values (PPV) of TSU and SUT in correctly identifying patients who relapsed at follow-up. The adjusted odds ratios (aOR) for predicting relapse from week 9–26 were 1.74 per point increase (95% CI = 1.05–2.89) for TSU and 1.59 (95% CI = 1.11–2.28) for SUT. The aORs for predicting relapse from week 9–52 were 2.41 (95% CI = 1.33–4.37) and 1.71 (95% CI = 1.14–2.56), respectively. Applying a cut-point of ≥3 on TSU resulted in SP = 97.1 and PPV = 70.0 in week 9–26, and SP = 98.8 and PPV = 90.0 in week 9–52. Applying a cut-point of ≥4 on SUT resulted in SP = 99.0 and PPV = 85.7 in week 9–26, and SP = 98.8 and PPV = 85.7 in week 9–52. Both TSU and SUT were valid predictors of long-term relapse in patients under smoking cessation treatment in primary care. These simple questions may be useful to implement in primary care.

**Trial registration:** Dutch Trial Register (NTR3067).

## Introduction

Tobacco smoking is a major risk factor for a range of highly prevalent diseases treated in primary care, including cardiovascular and respiratory disease^1^. After the age of 35, tobacco smokers lose an average of three months of life for every year of continued smoking and substantially increase their risk of chronic morbidity^2^. It is therefore vital that smokers stop smoking permanently as soon as possible and that every attempt to quit has the highest chance of success. As the majority of quit attempts ends with a relapse, preventing relapse is a top priority in smoking cessation treatment^3^.

In the context of primary care, where many smokers are treated for tobacco addiction, it would be very relevant to identify patients at high risk of relapse in order to target relapse prevention interventions efficiently, such as adding or extending pharmacological treatment^4^. However, a diagnostic tool for such a purpose would not only need to be valid but also very quick and easy to apply in order to be actually used by general practitioners.

The time spent with urges to smoke in the past 24 h and the strength of urges to smoke are two simple and short questions, which have been shown in a population study to predict the success of future quit attempts in current smokers^5^. An important advantage of these measures, compared with other measures of tobacco dependence (such as the widely used Fagerström Test for Cigarette Dependence^6,7^ and its abbreviated version, the Heaviness of Smoking Index^8^), is that they do not rely on measurements of cigarette consumption and can therefore also be used in ex-smokers, in particular in smokers currently trying to quit smoking. However, their usefulness in primary care has not been investigated so far.

We explored data from a randomised controlled smoking cessation trial in primary care^9^ to assess the validity of these two urges to smoke measures in predicting long-term relapse in patients who were abstinent from smoking for several weeks. Furthermore, we explored whether applying a cut-point on the rating of these urges would allow identifying patients at high risk of relapse with acceptable certainty.

## Results

### Study population

A total of 295 smoking patients were included in the trial, of which 180 (61%) were abstinent at week 9 after treatment initiation^9^. This subgroup formed the study population of the current investigation. By weeks 26 and 52, 42% (75/180) and 56% (100/180), respectively, had relapsed^9^.

The mean age of the study population was 51 years (standard deviation, SD = 12.0), and 52% (93/180) were female. The mean score was 1.63 (SD = 0.67) for TSU and 1.81 (SD = 0.90) for SUT. Furthermore, median scores were 2 (range = 1–5) for both measures, indicating that a majority of patients reported moderate urges to smoke a little of the time during the past 24 h.

### Predictors of relapse

Table 1 gives an overview of the unadjusted and adjusted odds ratios (OR) and corresponding 95% confidence intervals (CI) of different predictors of relapse during weeks 9–26 and 9–52. Both TSU and SUT were significantly associated with relapse during week 9–26 and week 9–52 in the unadjusted and adjusted models. TSU showed a stronger association than SUT and predicted relapse during week 9–52 (OR = 2.41 per point increase, 95% CI = 1.33–4.37) even better than relapse during week 9–26 (OR = 1.74 per point increase, 95% CI = 1.05–2.89). The baseline measurements of CDP, TTFC and HSI showed no association with the two endpoints.

**Table 1:** Predictors of relapse during week 9–26 and week 9–52.

Predictor	Relapse during week 9–26		Relapse during week 9–52	
	OR (95% CI)	aOR (95% CI)	OR (95% CI)	aOR (95% CI)
TSU in week 9	1.65* (1.03–2.63)	1.74* (1.05–2.89)	1.93* (1.17–3.18)	2.41** (1.33–4.37)
SUT in week 9	1.57* (1.11–2.21)	1.56* (1.07–2.28)	1.59* (1.11–2.28)	1.71* (1.14–2.56)
CDP at baseline	1.14 (0.78–1.66)	1.19 (0.77–1.83)	1.16 (0.79–1.69)	1.31 (0.85–2.03)
TTFC at baseline	0.92 (0.65–1.30)	0.76 (0.50–1.14)	0.80 (0.56–1.14)	0.73 (0.49–1.11)
HSI at baseline	1.01 (0.81–1.26)	0.96 (0.74–1.22)	0.96 (0.78–1.20)	0.98 (0.77–1.25)

*TSU* time spent with urges (1–6 = all of the time), *SUT* strength of urges to smoke (1–6 = extremely strong), *CPD* cigarettes per day (<10, 11–20, 21–30, and >31), *TTFC* time to first cigarette (within 5 min, 6–30, 31–60, and more than 60), *HSI* Heaviness of Smoking Index (0–6 = highest level of tobacco dependence), *aOR* odds ratio adjusted for the treatment group, health care centre, age, gender, education, income, level of self-efficacy, duration of a longest previous quit attempt, level of depression, level of anxiety, number of smokers in the social environment and alcohol misuse.

*p < 0.05, **p < 0.01

### Diagnostic parameters

The diagnostic parameters for all possible cut-points on TSU and SUT are presented in Supplementary Table 1. As expected, with increasing cut-point SP and PPV generally increased whereas SN decreased. When choosing a cut-point of ≥3 on TSU, 6% of patients (10/180) were classified as being at high risk of relapse (Table 2). Applying this cut-point resulted in SP = 97.1 and PPV = 70.0 for correctly identifying patients who relapsed during week 9–26, and SP = 98.8 and PPV = 90.0 in week 9–52. When choosing a cut-point of ≥4 on SUT, 4% of patients (7/180) were classified as being at high risk of relapse. Applying this cut-point resulted in SP = 99.0 and PPV = 85.7 for correctly identifying patients who relapsed in week 9–52, and SP = 98.8 and PPV = 85.7 in week 9–52.

**Table 2:** Diagnostic parameters for urges to smoke measures as predictors of relapse during week 9–26 and week 9–52.

Predictor	Week 9–26					Week 9–52				
	Relapsed (*N*)	Abstinent (*N*)	SN (%)	SP (%)	PPV^a^ (%)	Relapsed (*N*)	Abstinent (*N*)	SN (%)	SP (%)	PPV^b^ (%)
TSU <3	68	102	9.3	97.1	70.0	91	79	9.0	98.8	90.0
TSU ≥3	7	3				9	1			
SUT <4	69	104	8.0	99.0	85.7	94	79	6.0	98.8	85.7
SUT ≥4	6	1				6	1			

*TSU* time spent with urges (1–6 = all of the time). *SUT* strength of urges to smoke (1–6 = extremely strong). *SN* sensitivity. *SP* specificity.

^a^PPV positive predictive value given a 42% prevalence of relapse.

^b^PPV positive predictive value given a 56% prevalence of relapse.

## Discussion

Both TSU and SUT, measured after 9 weeks of abstinence in primary care patients receiving smoking cessation treatment, were valid predictors of long-term relapse after 26 and 52 weeks. Applying a cut-point of ≥3 on TSU and ≥4 on SUT resulted in a PPV of 90 and 86% after 52 weeks, respectively.

Many smoking cessation trials have reported measures of urges to smoke and other withdrawal symptoms, but we are not aware of a previous study exploring the use of urges to smoke measures in primary care patients receiving smoking cessation treatment, particularly exploring the idea of applying cut-points of these measures in order to use them as a diagnostic tool. However, our study has several limitations. Our analysis was explorative and not planned a priori, using data from a medium-size smoking cessation trial, which was originally designed to compare two behavioural treatments for smoking cessation alongside pharmacotherapy in primary care. Hence, our findings should be interpreted within this context. Both treatment groups received varenicline, a medication that works by reducing craving as a nicotine receptor partial agonist. This explains why only a minority of patients from our trial reported strong urges to smoke (a value above our chosen cut-points) at the time of the measurement. It may be that patients receiving behavioural treatment and varenicline relapse for different reasons than patients trying to quit with different aids or unaided. Our idea would therefore need to be tested in a pre-specified trial specifically designed to investigate the feasibility and effectiveness of using urges to smoke measures as a diagnostic tool in primary care and possibly other settings, preferably in smokers who try to quit unaided or with only minimal behavioural support. In such a trial, it would also be of interest to examine the completion and interpretation of the questions on urges during the patients’ consultation of the GP practice (contrary to our trial, where patients filled out a questionnaire at home). Our study does not answer the question of whether urges were a direct measure of relapse risk or perhaps only an indicator for different, underlying reasons^10^. Furthermore, it is well known that smokers perceive urges to smoke, which are generally high at the beginning of the quit attempt and decrease over time and that urges to smoke trajectories can vary considerably between smokers^11,12^. In our study, a measurement at one time point (after 9 weeks of abstinence; this time point was given by means of the original trial protocol) was predictive of long-term relapse, but the predictive validity and diagnostic value may be different and more relevant at different time points of abstinence and in smokers using different treatments or no treatment at all.

We conclude that the concept of using urges to smoke measures as a diagnostic tool for the management of smoking cessation treatment may be of relevance for primary care and possibly other health care settings. Both TSU and SUT are simple and short questions, which provide the GP (or other primary health care workers who provide smoking cessation support) with an immediate result, which makes them potentially useful for implementation during patient-physician consultations in primary care. Using urges to smoke measures as a diagnostic tool may be particularly interesting in patients consulting the GP practice who are in the process of trying to quit unaided, because this is the largest group of smokers and because the GP can make a strong case to recommend the use of evidence-based smoking cessation treatments in smokers at highest risk of relapse^4,13^. The GP could then also investigate underlying causes of urges lying in the social environment of the patient (e.g., a smoking partner or smoking at the workplace) and discuss possible solutions to reduce the risk of relapse (e.g., by making a shared decision to use or extend the duration of smoking cessation pharmacotherapy or by recommending a more intensive behavioural support programme). However, as noted before, verification and elaboration of our findings in future studies are necessary.

## Methods

### Study design

Our trial compared the effectiveness of intensive practice nurse counselling versus brief general practitioner (GP) advice, both combined with varenicline, for smoking cessation in primary care patients. We conducted the multi-site trial in a network of ten primary health care centres in the Netherlands in the period between October 2011 and July 2014. Patients provided written informed consent to take part in the study. The trial had obtained ethical approval (NL30057.068.09/METC 09-03-075) and had been registered in the Dutch Trial Register (NTR3067). Details of the study design were published in a protocol^14^.

In brief, we recruited patients who smoked daily and had no contraindications for varenicline. Patients were individually randomised to either intensive smoking cessation counselling by a trained PN or brief advice to quit by a GP from their health care centre. In addition, all patients received a 12-week course of varenicline according to the usual dosing scheme. The primary endpoint was prolonged smoking abstinence from week 9 through week 26 after treatment initiation, biochemically confirmed by exhaled carbon monoxide (CO <10 ppm), as defined by the Russell Standard^15^. A secondary endpoint was abstinence from week 9 to 52.

### Measurement of predictors

Urges to smoke were measured at week 9 with two items taken from the Mood and Physical Symptoms Scale (see Supplementary Note 1 for the Dutch translation that was applied)^16^. Time spent with urges to smoke (TSU) was measured by asking: “How much of the time have you felt the urge to smoke in the past 24 h?”, with response options “not at all” (coded 1), “a little of the time” (2), “some of the time” (3), “a lot of the time” (4), “almost all of the time” (5) and “all of the time” (6). Strength of urges to smoke (SUT) was measured by asking: “In general, how strong have the urges to smoke been?”, with the response options “no urges” (1), “slight” (2), “moderate” (3), “strong” (4), “very strong” (5) and “extremely strong” (6). In a population survey, both items showed to be valid predictors of the success of future quit attempts in current smokers^5^.

Only at baseline, we also measured the number of cigarettes per day (CPD: ≤10 (coded 0), 11–20 (1), 21–30 (2), ≥31 (3)) and time to first cigarette (TTFC: within 5 min (coded 3), 6–30 min (2), 31–60 min (1), more than 60 min (0)). The sum of these items is the Heaviness of Smoking Index (HSI; range 0–6)^17^. Furthermore, we measured the following covariates: age, gender, education, income, self-efficacy, duration of a longest previous quit attempt in years, depression, anxiety, number of smokers in the social environment and alcohol misuse^9^.

### Statistical analyses

We used logistic regression models to assess whether TSU and SUT (as continuous variables) predicted relapse during week 9–26 and week 9–52, both unadjusted as well as adjusted for the above-mentioned covariates in addition to the treatment group and health care centre. For comparison, we ran these models with the baseline measures of CPD, TTFC and HSI as predictor variables. We then dichotomised TSU and SUT based on all possible cut-points in order to calculate the sensitivity (SP), specificity (SP) and positive predictive values (PPV) of TSU and SUT in correctly identifying patients who relapsed during week 9–26 and week 9–52. Finally, we suggested a cut-point for TSU and SUT with sufficiently high SP to detect relapse^18^.

### Reporting Summary

Further information on research design is available in the Nature Research Reporting Summary linked to this article.

## Supplementary information

Supplementary Information

Reporting Summary

## Data Availability

The dataset analysed during the current study is available from the corresponding author on reasonable request.
