# Supplementary material for: Validity of urges to smoke measures in predicting smoking relapse during treatment in primary care
Source: NPJ Prim Care Respir Med. 2021 Dec 9;31:48. doi: 10.1038/s41533-021-00259-3 (PMC8660873; doi:10.1038/s41533-021-00259-3)
Supplement: Supplementary file 1 — Supplementary Information [file 41533_2021_259_MOESM1_ESM.pdf]

SUPPLEMENTARY INFORMATION

Supplementary Note 1: Original English and translated Dutch versions of the two urges to smoke measures; time spent with urges to smoke (TSU) and strength of urges to smoke (SUT)

TSU

	**Original English version**[1]	**Dutch translation**
	How much of the time have you felt the urge to smoke in the past 24 hours?	Hoe vaak heeft u in de afgelopen 24 uur de drang gevoeld om te roken?
6.	all of the time	continue
5.	almost all of the time	bijna de hele tijd
4.	a lot of the time	een groot deel van de tijd
3.	some of the time	een redelijk deel van de tijd
2.	a little of the time	een klein deel van de tijd
1.	not at all	totaal niet

SUT

	**Original English version**[1]	**Dutch translation**
	In general, how strong have the urges to smoke been?	Hoe sterk was deze drang?
6.	extremely strong	extreem sterk
5.	very strong	zeer sterk
4.	strong	sterk
3.	moderate	matig
2.	slight	licht
1.	no urges	geen drang

[1] Fidler JA, Shahab L, West R. Strength of urges to smoke as a measure of severity of cigarette dependence: comparison with the Fagerstrom Test for Nicotine Dependence and its components. Addiction. 2011;106(3):631-8

Supplementary Table 1: Diagnostic parameters for urges to smoke measures as predictors of relapse during week 9-26 and week 9-52, including all possible cut-points

Predictor	Week 9-26					Week 9-52				
	**Relapsed** (N)	**Abstinent** (N)	**SN** (%)	**SP** (%)	**PPV**[a] (%)	**Relapsed** (N)	**Abstinent** (N)	**SN** (%)	**SP** (%)	**PPV**[b] (%)
TSU ≥1	75	105	100	0	41.7	100	80	100	0	55.6
TSU ≥2	48	52	64.0	50.4	48.0	62	38	62.0	52.5	62.0
TSU ≥3	7	3	9.3	97.1	70.0	9	1	9.0	98.8	90.0
TSU ≥4	1	2	1.3	98.1	33.3	3	0	3.0	100	100
TSU ≥5	1	0	1.3	100	100	1	0	1.0	100	100
TSU ≥6	0	0	-	-	-	0	0	-	-	-
SUT ≥1	75	105	100	0	41.7	100	80	100	0	47.6
SUT ≥2	47	51	62.7	51.4	48.0	61	37	61.0	53.8	56.7
SUT ≥3	21	17	28.0	83.8	55.3	27	11	27.0	86.3	67.7
SUT ≥4	6	1	8.0	99.0	85.7	6	1	6.0	98.8	85.7
SUT ≥5	2	0	2.7	100	100	2	0	2.0	100	100
SUT ≥6	0	0	-	-	-	0	0	-	-	-

TSU=time spent with urges (1–6=all of the time). SUT=strength of urges to smoke (1–6=extremely strong). SN=sensitivity. SP=specificity. [a]PPV=positive predictive value given a 42% prevalence of relapse. [b]PPV=positive predictive value given a 56% prevalence of relapse.
